# Whole-Body Lifetime Occupational Lead Exposure and Risk of Parkinson’s Disease

**DOI:** 10.1289/ehp.9102

**Published:** 2006-08-18

**Authors:** Steven Coon, Azadeh Stark, Edward Peterson, Aime Gloi, Gene Kortsha, Joel Pounds, David Chettle, Jay Gorell

**Affiliations:** 1 Department of Biostatistics and Research Epidemiology and; 2 Department of Pathology, Henry Ford Health System, Detroit, Michigan, USA; 3 Department of Radiology, St. Vincent Hospital, Green Bay, Wisconsin, USA; 4 Department of Neurology, Henry Ford Health System, Detroit, Michigan, USA; 5 Department of Molecular Biosciences, Pacific Northwest National Laboratory, Richland, Washington, USA; 6 Medical Physics and Applied Radiation Sciences Department, McMaster University, Hamilton, Ontario, Canada

**Keywords:** case control, chronic toxicity, K-X-ray fluorescence, lead exposure, neurodegeneration, occupational exposure, Parkinson’s disease

## Abstract

**Background:**

Several epidemiologic studies have suggested an association between Parkinson’s disease (PD) and exposure to heavy metals using subjective exposure measurements.

**Objectives:**

We investigated the association between objective chronic occupational lead exposure and the risk of PD.

**Methods:**

We enrolled 121 PD patients and 414 age-, sex-, and race-, frequency-matched controls in a case–control study. As an indicator of chronic Pb exposure, we measured concentrations of tibial and calcaneal bone Pb stores using ^109^Cadmium excited K-series X-ray fluorescence. As an indicator of recent exposure, we measured blood Pb concentration. We collected occupational data on participants from 18 years of age until the age at enrollment, and an industrial hygienist determined the duration and intensity of environmental Pb exposure. We employed physiologically based pharmacokinetic modeling to combine these data, and we estimated whole-body lifetime Pb exposures for each individual. Logistic regression analysis produced estimates of PD risk by quartile of lifetime Pb exposure.

**Results:**

Risk of PD was elevated by > 2-fold [odds ratio = 2.27 (95% confidence interval, 1.13–4.55); *p* = 0.021] for individuals in the highest quartile for lifetime lead exposure relative to the lowest quartile, adjusting for age, sex, race, smoking history, and coffee and alcohol consumption. The associated risk of PD for the second and third quartiles were elevated but not statistically significant at the α = 0.05 level.

**Conclusions:**

These results provide an objective measure of chronic Pb exposure and confirm our earlier findings that occupational exposure to Pb is a risk factor for PD.

Parkinson’s disease (PD) is a neurologic movement disorder in which neurons of the substantia nigra, which are responsible for dopamine production, attenuate in number or become functionally impaired. Resultant symptoms include resting tremor in the extremities and head, limb and/or trunk rigidity, bradykinesia, and postural instability. Although the primary cause of the destruction or failure of substantia nigra cells is unknown, mounting evidence exists for both environmental and genetic determinants. A growing body of evidence suggests that heavy metal cations stimulate free radical formation in the brain and can lead to neurodegeneration via peroxidative damage to the cell membrane (Sandhir et al. 1994). Increasing levels of heavy metal cations stimulate the conformational changes that can lead to fibrillation of recombinant α-synuclein. The aggregation and fillibration of α-synuclein provoked by the presence of heavy metal cations could directly cause the intracellular protein inclusions that are observed in the substantia nigra of PD patients (Uversky et al. 2001). Results from several epidemiologic studies further support an association between PD and exposure to heavy metals (Aquilonius and Hartvig 1986; Rybicki et al. 1993; Tanner et al. 1989). Our previous results demonstrated a 2-fold increase in the risk of PD among workers with > 20 years of occupational exposure to Pb. Associations of such chronic occupational exposure to combinations of Pb–iron and Pb–copper were even more robust (Gorell et al. 1997).

Although several studies have examined the relationship between blood Pb levels and environmental Pb exposure, results from blood Pb analysis are not noteworthy. Research into long-term or distant past Pb exposures is confounded by the body’s ability to purge metals quickly from the blood stream. Because the half-life of Pb in blood is approximately 1 month, blood Pb levels only reveal if the individual has experienced a relatively current exposure to environmental Pb. Thus, blood Pb levels indicate only acute and recent exposures to Pb rather than representing a chronic and/or extended exposure history. Rather than within circulating blood, the main repository for chronic Pb stores in the body is within bone (Hu et al. 1998). Pb is stored in the bone by replacing calcium of the hydroxyapatite crystals of the bone mineral. Through a continual process of bone remodeling, synthesis, and reabsorption, Pb is released into the blood stream and circulates throughout the body. In the brain, Pb diffuses easily across the blood brain barrier and binds to sulphydryl groups, resulting in Pb neurotoxicity that leads the neurodegeneration via intracellular oxidative damage (Bradbury and Deane 1993; Popenoe and Schmaeler 1979; Quinlan et al. 1988; Rossman 2006; Sandhir et al. 1994).

Additionally, while the half-life of Pb in blood is very short, the half-life of Pb in bone is measured in years and decades, depending on the type of bone [the hard bone of the tibia (half-life of approximately 20 years) releases Pb more slowly over time than the soft, spongy bone of the calcaneus (half-life of ≤ 10 years)] and the individual’s metabolic rate of leaching and clearance (Patterson 1980). Measuring Pb concentration in bone using K-shell X-ray fluorescence (K-XRF) provides a proxy for current whole-body Pb content, independent of whether the exposure is current (Hu et al. 1991; Todd and Chettle 1994). Because K-XRF measurements estimate the current level of blood stores in the body, they can be used to construct an indirect assessment of an individual’s past exposures to Pb. If the exposure to Pb was experienced at some time in the past, the individual’s body will have cleared some amount of Pb between the last period of exposure and the time of the K-XRF measurement. This clearance rate of Pb from bone is predictable and can be used in a physiologically based pharmacokinetic (PBPK) model to estimate the level of Pb in the body at the actual time of the exposure. Kinetic modeling allows for a more complete picture of the individual’s lifetime occupational Pb exposure by combining current body stores, as detected by bone Pb levels; current exposures, as indicated by blood Pb levels; and both current and past environmental exposures, as reported by the individual and qualified by industrial hygienist assessment to assess the timing and intensity of the exposure (Leggett 1993).

In this study, we further evaluated the risk of PD in response to the total body Pb burden. To accomplish this, we conducted a case–control study with the following objectives: *a*) to integrate historical exposure information with current measurements of cortical and trabecular bone Pb obtained by current K-XRF and blood Pb; *b*) to reconstruct the Pb exposure history using PBPK models; and *c*) to use the resultant estimate of the net body Pb burden over time (dose) to calculate a quantitative relationship between the lifetime exposure and the risk of PD.

## Materials and Methods

### Setting and study population

**Figure 1:**
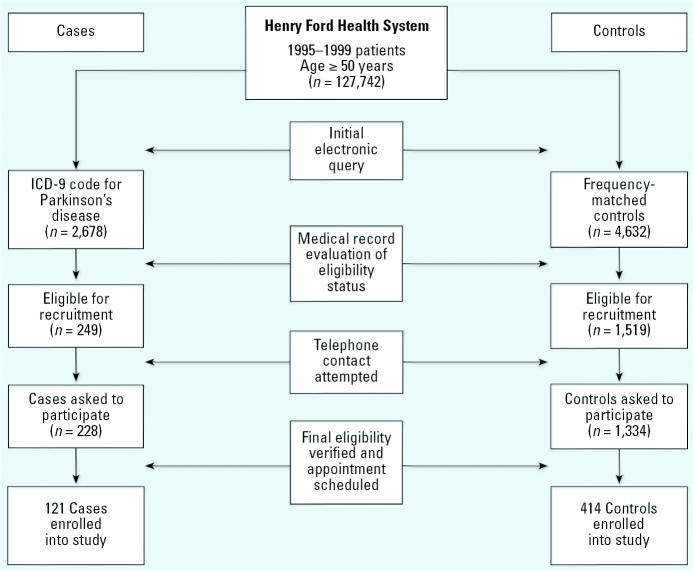
Diagram of case and control selection, recruitment, and enrollment.

Study participants were patients who had received their primary health care services between 1995 and 1999 from the Henry Ford Health System (HFHS), the second largest health care provider in southeastern Michigan. We identified potential study participants ≥ 50 years of age from a database containing data for 127,742 patients using the internal Corporate Data Store (CDS) computerized billing system within HFHS (Figure 1). We implemented a multistep screening process for the ascertainment of PD diagnosis. First, we used the *International Classification of Disease, Ninth Revision* (ICD-9; *World Health Organization* 1978) codes 332 and 332.0 to identify potential cases from the CDS; we identified a total of 2,678 potential cases. In the second step of screening, we reviewed electronic and/or hard copies of medical records of the 2,678 individuals for a diagnosis of idiopathic PD within the previous 5 years. Of these, a total of 249 individuals had confirmed clinical diagnoses of PD. We randomly identified potential controls from the CDS and frequency-matched them on sex, race, and age at the time diagnosis of PD (± 5 years). Frequency-matching reduces the primary effects of these variables in the analysis phase. We reviewed the medical records of potential cases to exclude patients with secondary Parkinsonism, stroke-induced Parkinsonism, and Parkinsonism as a result of head injury, brain tumor, encephalitis, neuroleptic use, carbon monoxide intoxication, Huntington’s disease, Wilson’s disease, essential or intention tremor, dementia, verbal aphasia, seizure disorder, psychosis or depression, substance abuse; we also excluded patients with hydrocephalus. One of the investigators (J.G.) and two registered nurses in the Department of Neurology reviewed all of the medical records and made the final screening eligibility judgment.

We mailed a recruitment packet containing a letter explaining the purpose of the study, a one-page sheet of frequently asked questions about PD and the research study, and a brochure describing the procedure of bone Pb measurement using K-XRF to a total of 249 cases and 1,519 potential control volunteers. A week after mailing, trained interviewers made follow-up telephone calls and invited the potential participants to participate in the study. Those individuals who agreed to do so underwent a telephone-administered shortened version of the Mini Mental State Exam (MMSE; Roccaforte et al. 1992) as the final step in the screening process. A total of 228 eligible cases and 1,334 eligible controls were asked to participate in the study; 121 cases (53.1%) and 414 controls (31.0%) agreed and were enrolled in the study. Participants were scheduled for a clinic visit during which they were administered the full version of the MMSE and the K-XRF procedure, had blood drawn for laboratory analysis of Pb, and were interviewed for potential occupational exposure to Pb and for other possible risk factors for PD. To rule out patients with dementia, all final participants had a full MMSE score ≥ 24 (American Psychiatric Association 1994; Anthony et al. 1982; Folstein et al. 1975). Participants gave written informed consent and were minimally compensated, and the study was approved by the institutional review board within the HFHS.

### Pb exposure assessment

During a face-to-face interview using a modified version of the Pb exposure questionnaire instrument of Kosnett et al. (1994), study participants described the types and duration of occupations held throughout their adult life (age 18 to present). One of the investigators (G.K.), an industrial hygienist, blinded to the status of the study participant, ranked the probability of Pb exposure and the intensity of exposure as high, medium, or low for each occupation lasting longer than 6 months. High-probability exposures occurred when the activity performance exceeded 90% of the work period and involved direct handling of Pb-containing materials, or the liberation of Pb-containing fumes, dusts, or liquids in the vicinity of the subject. Moderate probabilities of Pb exposure (10–90% of the time) and low probabilities (< 10% of the time) were associated with “occasional” (moderate) or “rare or never” (low) contact with leaded materials or Pb-containing fumes, dusts, or liquids. The intensity of exposure was rated high if the probability of exposure was also high and the permissible exposure level (PEL) standard (Occupational Safety and Health Administration 1998) was exceeded > 50% of the time, giving a high score using the paradigm of Sharp et al. (1991). Moderate intensities of exposure were associated with 10–10% of the PEL and a moderate level by Sharp et al. (1991), and low intensities were < 10% of the PEL with a low exposure score, according to Sharp et al. (1991). We then combined probability and intensity scores with duration for each occupation, so each subject had a cumulative Pb exposure history over time from age 18 to the time of recruitment.

### Bone Pb measurement by K-XRF

We performed K-XRF measurement of bone Pb on each subject during 40 min of exposure to the left tibia and calcaneus to a sealed 1.1 GBq (30 mCi) ^109^Cd source, placed in a commercial detector (Canberra Industries, Meriden, CT), 2 cm from each measured bone. The reflected 88 keV γ-rays were amplified and digitized, and we collected the energy spectrum on a multichannel analyzer. We determined the amplitudes of α1 and β1 K-series X rays and their ratios to the amplitude of the coherent scatter peak for each human bone spectrum and evaluated calibration lines generated from a set of 10 standard Pb-doped plaster-of-Paris phantoms with Pb concentrations ranging from 0 to 200 μg/g. Using a nonlinear chi-square minimization procedure, we fit models of the spectral features to each individual’s data (Bevington 1969; Marquardt 1963). The amplitudes of the Pb X rays and of the coherently scattered 88 keV γ-rays were extracted for further analysis. We based quantity assessment on independent estimates of the amplitudes of α1 and β1 K-band X rays and their ratios to the amplitude of the coherent scatter peak, giving two calibration lines corresponding to α and βX rays. The same two ratios were determined for each human bone spectrum and evaluated against the appropriate calibration lines, making due allowance for the difference in coherent scattering between bone and plaster of Paris. We calculated the result for an individual subject as the inverse variance of the weighted mean of the two ratios. At the time of K-XRF measurement, we drew 5 mL venous blood from each subject for determination of blood Pb levels. Samples were analyzed by atomic absorption spectroscopy at Henry Ford Hospital (Detroit, MI) (Miller et al. 1987).

### PBPK modeling of lifetime Pb exposure

We integrated Pb exposure duration and intensity data with current measures of blood and skeletal Pb concentrations using the International Commission for Radiation Protection (ICRP) PBPK model (Leggett 1993). We developed an algorithm to convert exposure questionnaire information objectively for use in biokinetic modeling. Each occupational activity that involved risk of Pb exposure was assigned the probability and intensity scale values of the activity (low, medium, and high), converted to 1, 2, or 3, respectively. The daily Pb input to the model from a particular activity was then determined using the following formula:


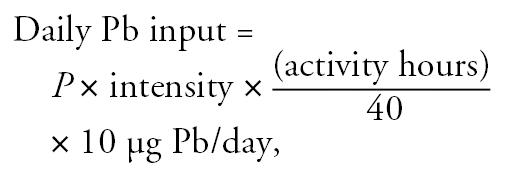


where *P* is the probability of Pb exposure because of job classification; “intensity” is the intensity of exposure; and “activity hours” is the number of hours devoted to the activity per 40-hr work week. The value of 10 μg Pb/day was derived from the assumption that an individual working 40 hr/week in a “high probability/high intensity” exposure activity should have a blood Pb level just above 40 μg/dL (Patterson 1980). A calculation of 3 × 3 × (40/40) × 10 = 90 μg/dL gave sufficient daily Pb input above the baseline to result in blood Pb levels of 42 μg/dL during this activity. We then calculated the total lifetime exposure for each individual by adding the Pb exposure questionnaire-derived values for each occupation plus the baseline Pb input at that subject’s age at enrollment. Next, we incorporated age-dependent changes in bone remodeling rates and skeletal mass above the age of 60 years and scaled the bio-kinetic model to an individual subject’s body weight, rather than to the commonly used 70-kg reference man (ICRP 1995).

We adjusted the intensity of Pb exposure (micrograms of Pb per day) in the model for the current blood Pb level, and for trabecular (calcaneus) and cortical (tibia) bone Pb concentration to minimize the model fit error using the formula


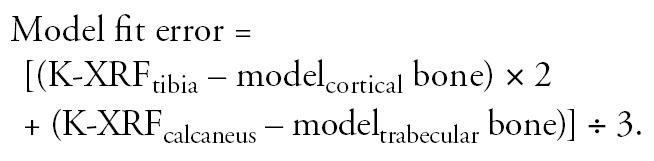


This calculation weights the model fitting to the K-XRF measurement of the tibia (cortical bone) because of the longer half-life of Pb in cortical bone and because the error of K-XRF measurement is smaller than for trabecular bone. The use of this equation provides an objective description of the model’s fit to limited data. Input K-XRF estimates of bone Pb ranged from 4.5 to 54.4 μg/g (micrograms of Pb per gram of bone) with a mean ± SD of 12.5 ± 7.8 μg/g for tibial measurements and from 9.3 to 64.4 μg/g with a mean of 20.5 ± 10.2 μg/g for calcaneal measurements. The resulting model fit error ranged from −37.5 to 18.5 μg/g and produced a mean error of −4.0 ± 6.7 μg/g.

### Statistical methods

To assess the distribution of exposures among case and control participants, we used chi-square analysis. To assess the relationship of Pb exposure to the presence of PD we used multiple logistic regression techniques, using the presence or absence of PD as the dependent variable. Odds ratios (ORs) and their associated 95% confidence intervals (CIs) were calculated. For all models a *p*-value < 0.05 was considered statistically significant. For Pb exposure, we calculated the whole-body lifetime Pb exposure as described above and categorized the participants into four quartiles based on the mean exposure per year for the control group: first quartile [0–40.04 μg/g (micrograms Pb storage per gram of bone)], second (40.05–57.43 μg/g), third (57.44–80.80 μg/g), and fourth (≥ 80.81 μg/g). Logistic regression analysis was run with three indicator variables and the first quartile of exposure serving as the reference group. The range of whole-body lifetime Pb exposure measurements was skewed by several large exposure values, making the data difficult to model as a continuous variable. A log-transform of the data modeled well, and these results are also included below.

Results of the K-XRF measurements were also examined via logistic regression using either tibia or calcaneus Pb exposure as the independent variable to be associated with PD. Tibia bone Pb exposure was categorized into quartiles based on the exposure of the control group as follows: first (0–5.91 μg/g), second (5.92–10.40 μg/g), third (10.41–15.50 μg/g), and fourth (≥ 15.51 μg/g). Finally, calcaneus bone Pb exposure was categorized into quartiles based on the exposure of the control group as follows: first (0–11.70 μg/g), second (11.71–19.07 μg/g), third (19.08–25.28 μg/g), and fourth (≥ 25.29 μg/g).

In adjusted logistic regression models, we included the matching covariates of age, sex, and race. Although we modeled age as a continuous variable, sex and race were both included as dichotomous variables of male/female and white/nonwhite, respectively. Because smoking history, coffee consumption, and alcohol consumption have been reported on extensively as effect modifiers in determining PD risk, we also included these factors in adjusted models. We calculated pack-years of smoking history (i.e., packs of cigarettes per day multiplied by years smoked), and categorized smokers into three groups: none (0 pack-years), mild to moderate (> 0 to 30 pack-years), and heavy (> 30 pack-years). The analysis thus ran two indicator variables, with nonsmokers serving as the reference group. We defined “coffee consumption history” as the number of coffee-years, where a “coffee-year” was defined as one cup of coffee per day for 1 year. We categorized coffee drinkers, using the median coffee consumption for the control group, as low (> 0–112 coffee-years) and high (> 112 coffee-years). Two indicator variables were used in the logistic regression analysis, and “no coffee consumption” served as the reference group. For alcoholic consumption, we defined a “drink-year” as the intake of one “drink” of alcohol per day for 1 year. We defined a “drink” of alcohol as follows: liquor (30 mL), one can of beer (360 mL), or one glass of wine (120 mL). We classified participants as nondrinkers (0 drink-years), mild to moderate drinkers (> 0–10 drink-years), or heavy drinkers (> 10 drink-years). Two indicator variables were used in the logistic regression analysis and nondrinkers served as the reference group.

## Results

We enrolled 535 nondemented men and women ≥ 50 years of age. The average age of participants in the study was 69.9 ± 8.2 years and did not differ from nonparticipants (69.7 ± 9.1; *p* = 0.69, Student’s *t*-test). Also, race did not differ significantly between participants (85.8% white) and nonparticipants (83.2% white; *p* = 0.19, Mantel-Haenszel chi-square). Sex, however, did differ significantly between participants (56.6% male) and nonparticipants (45.7% male; *p* = 0.001, Mantel-Haenszel chi-square). This difference resulted from the combination of both a higher incidence of PD within males and our inability to recruit healthy males as effectively as healthy females for participation.

Of the 535 participants, 121 had a verified idiopathic diagnosis of PD, and 414 were healthy age-, sex-, and race-, frequency-matched controls (Table 1). This included 303 (56.6%) males and 232 (43.4%) females, 460 (86.0%) whites and 75 (14.0%) nonwhites. Even with a recruitment protocol designed for frequency matching, we observed a statistical difference for age and race. Individuals ≥ 70 years of age (*p* = 0.001) and whites (*p* = 0.038) were underrepresented among healthy controls compared with PD cases. Cases had an average age of 72.3 years (median 73.1 years) and controls had an average age of 69.7 years (median 70.0 years). Although males were also somewhat underrepresented among healthy controls, the difference from cases did not reach statistical significance. To account for the difficulty in balancing recruitment among these groups, the final logistic regression models included adjustment for these variables. Distributions of the covariates for smoking history, coffee consumption, and alcohol consumption are shown in Table 1. Patients with PD were more likely to be non-smokers or to be lighter smokers than healthy controls (*p* = 0.002). Controls were nearly twice as likely to be heavy smokers (> 30 pack-year history). PD patients were also less likely to be coffee drinkers than controls (*p* = 0.007). No difference in alcoholic consumption was observed between patients with PD and healthy control volunteers.

Participants were categorized into quartiles of exposures individually for categories of whole-body lifetime Pb exposure, K-XRF calcaneal bone Pb measurement, and K-XRF tibial bone Pb measurement, as described in “Materials and Methods.” Among patients with PD, whole-body Pb concentrations were lowest in the first quartile (14.9%) and highest in the fourth quartile (32.2%), yielding a positive indication for an association between Pb exposure and PD (*p*-trend = 0.036). Delegation of tibial K-XRF measurements into quartiles also reveals a significant trend from the first to fourth quartile (*p*-trend = 0.012). A statistically significant trend is not seen, however, when applied to quartiles of calcaneal K-XRF measurements (*p*-trend = 0.275).

Results from logistic regression modeling of quartiles of whole-body lifetime Pb exposure showed a statistically significant elevation of risk of PD for those in the fourth (highest exposure) quartile compared with those in the first (lowest exposure) quartile (Table 2). The estimated OR, 95% CI, and *p*-value indicate an elevated risk of PD for those in the fourth quartile, adjusting for age, sex, race, smoking, and coffee and alcohol consumption [OR = 2.27 (95% CI, 1.13–4.55); *p* = 0.021]. Individuals who experienced the highest quartile of exposure were twice as likely to have PD than those in the lowest quartile of exposure. The risk estimates for tibial and calcaneal K-XRF measurements of bone Pb concentrations alone (without incorporation of duration and intensity of occupational exposure data or blood Pb levels) were elevated in increasing quartiles of exposure, but these data did not reach statistical significance. In the adjusted lifetime Pb exposure model, moderate [OR = 0.58 (95% CI, 0.35–0.95); *p* = 0.029] and heavy [OR = 0.33 (95% CI, 0.17–0.65); *p* = 0.001] levels of smoking were associated with reduced risks of PD. Also, mild-to-moderate [OR = 0.41 (95% CI, 0.22–0.75); *p* = 0.004] and heavy [OR = 0.36 (95% CI, 0.19–0.68); *p* = 0.002] coffee drinkers displayed a reduced risk of PD as well. Neither mild-to-moderate nor heavy alcohol consumption was significantly associated with PD risk.

We then analyzed the log transformation of the continuous lifetime Pb exposure data by logistic regression. Adjusted, whole-body lifetime Pb remained significant [OR = 1.74 (95% CI, 1.10–2.75); *p* = 0.018], whereas K-XRF measurements of tibia [OR = 1.47 (95% CI, 0.99–2.20); *p* = 0.059] and calcaneal [OR = 1.53 (95% CI, 0.93–2.54); *p* = 0.097] did not reach statistical significance. Stratified analysis pointed to a stronger association between lifetime Pb exposure and PD risk for those older than [OR = 2.17 (95% CI, 1.16–4.06); *p* = 0.015] compared with those younger than [OR = 1.30 (95% CI, 0.62–2.70); *p* = 0.49] the median age (70.7 years), but testing for an interaction did not show a statistically significant difference between the odds ratios (*p* = 0.24). Similarly, stratified analysis by sex suggested that the association of lifetime Pb exposure to PD risk was higher in females [OR = 2.23 (95% CI, 1.05–4.76); *p* = 0.038] than in males [OR = 1.53 (95% CI, 0.83–2.80); *p* = 0.17], but an interaction test showed that this difference was not statistically significant (*p* = 0.50).

Independent of PD diagnosis, age, sex, smoking, coffee, and alcohol were associated with whole-body lifetime Pb exposure (data not shown) but race was not. Even so, the estimate of PD risk changed very little before and after adjustment for these covariates. The logistic model for whole-body lifetime Pb exposure had 99% and 98% power to detect a difference in the unadjusted and adjusted models, respectively. The logistic model for tibial K-XRF measurement of Pb exposure had 92% and 72% power to detect a difference in the unadjusted and adjusted models, respectively. The logistic model for tibial K-XRF measurement of Pb exposure had 57% and 79% power to detect a difference in the unadjusted and adjusted models, respectively.

## Discussion

Occupational exposure to heavy metals is associated with the risk of PD (Gorell et al. 1997). Assessment of blood Pb concentration as a proxy for chronic or distant past exposure is inconclusive because of the body’s ability to purge metals quickly from the blood stream. We used K-XRF technology to improve the assessment of chronic Pb exposure by measuring long-term Pb stores in the body. The use of K-XRF represents an advancement in methodology beyond our earlier work, which subjectively assessed occupational exposure to Pb by an experienced industrial hygienist (Todd and Chettle 1994; Uversky et al. 2001). Additionally, we employed PBPK modeling, which combined the bone Pb concentration with occupational history and blood Pb concentration to estimate whole-body lifetime Pb burden. Our findings show that higher lifetime exposure to Pb is associated with risk of PD and confirm previous findings that prolonged occupational exposure to Pb increases the risk of PD by more than 2-fold (Gorell et al. 1997). This study agrees with the findings of several other epidemiologic studies and further supports an association between PD and exposure to heavy metals (Aquilonius and Hartvig 1986; Rybicki et al. 1993; Tanner et al. 1989).

Recall bias is a primary source for error in the study of occupational exposure to heavy metals. This can manifest as either omission of exposure or exaggeration of duration and/or intensity of exposure. In an effort to reduce recall bias, we supplemented the participant’s self-report with the experience of the industrial hygienist to determine if Pb was used in a specific occupation. Also, by focusing primarily on occupational exposures—verifiable by the industrial hygienist—rather than environmental and recreational exposure, we further reduced the likelihood of spurious findings. Another limitation of the present study involves the failure to accurately match cases and controls on age and sex. Individuals ≥70 years of age were underrepresented in the control group compared to the cases. Adjustment for age in the logistic model that tested for the association of Pb exposure on PD risk did appreciably alter the effect size or the significance level. The results of the stratified analysis point to age as being an effect modifier for the association of Pb exposure to PD risk, but not the source of the effect itself. Similarly, males were underrepresented in the control group compared to cases. Here also, the adjustment for sex did not significantly change the effect size or significance level of the association between Pb exposure and risk of PD. The stratified analysis showed elevated ORs in both males and females that, while not significantly different, were stronger in females. More participants than nonparticipants were male, and this may have resulted in a further source of error. Because the association between Pb exposure and PD was stronger in females, it might be expected that the overall association in the general population would be stronger than that reported above.

This study supports the hypothesis that Pb plays a role in the etiology of PD in exposed individuals. Although the biochemical mechanism of Pb neurotoxicity is not completely understood, a growing body of evidence suggests that metal cations of Pb, iron, and aluminum stimulate free radical formation, which results in neurodegeneration via peroxidative damage to the cell wall. Sandhir et al. (1994) observed that increasing Pb concentration in rat brain produces heightened levels of lipid peroxidation and decreased activity of neuroprotective antioxidant enzymes and acetylcholinesterase. The authors suggested that lipid peroxidation eventually can lead to neuronal cell death through deterioration of the cell membrane. Uversky et al. (2001), using *in vitro* models of human brain cells, found that increasing levels of heavy metal cations stimulate the conformational changes that can lead to fibrillation of recombinant α-synuclein. The authors argued that the aggregation and fibrillation of α-synuclein provoked by the presence of heavy metal cations could directly cause the intracellular protein inclusions that are observed in the substantia nigra of PD patients. Quinlan et al. (1988) observed that although Pb ions alone did not induce peroxidation, they did accelerate the rate of peroxidation caused by iron ions. The present study provides additional objective evidence to support the hypothesis that long-term exposure to heavy metals, such as Pb, contributes to the accumulation of peroxidative damage and neurodegenerative cell death that is observed in PD.

## Figures and Tables

**Table 1:** Distribution of matched variables and potential confounders for cases (*n* = 121) and controls (*n* = 414).

Matching variable	Cases [no. (%)]	Controls [no. (%)]	*p*-Value^a^
Age (years)			
50–59	9 (7.4)	60 (14.5)	0.001*
60–69	27 (22.3)	147 (35.5)	
70–79	63 (52.1)	166 (40.1)	
≥80	22 (18.2)	41 (9.9)	
Sex			
Male	76 (62.8)	227 (54.8)	0.119
Female	45 (37.2)	187 (45.2)	
Race			
White	111 (91.7)	349 (84.3)	0.038*
Nonwhite	10 (8.3)	65 (15.7)	
Smoking			
None	70 (57.9)	166 (40.1)	0.002*
Mild to moderate	36 (29.8)	158 (38.2)	
Heavy	15 (12.4)	90 (21.7)	
Coffee			
None	30 (24.8)	53 (13.0)	0.007*
Mild to moderate	46 (38.0)	180 (44.1)	
Heavy	45 (37.2)	175 (42.9)	
Alcohol			
None	20 (16.5)	53 (13.0)	0.544
Mild to moderate	45 (37.2)	168 (41.1)	
Heavy	56 (46.3)	188 (46.0)	

a Chi-square.

* Statistically significant.

**Table 2:** Risk of PD by whole-body lifetime Pb exposure and K-XRF point measurements of lead exposure.

	Unadjusted		Adjusted^a^	
Variable	OR (95% CI)	*p*-Value	OR (95% CI)	*p*-Value
Whole body				
Second quartile	1.87 (0.99–3.52)	0.052	1.90 (0.97–3.71)	0.060
Third quartile	1.67 (0.87–3.18)	0.121	1.71 (0.86–3.41)	0.125
Fourth quartile	2.15 (1.15–4.00)	0.016*	2.27 (1.13–4.55)	0.021*
Tibia				
Second quartile	0.90 (0.47–1.73)	0.762	0.87 (0.43–1.75)	0.691
Third quartile	1.52 (0.84–2.75)	0.165	1.33 (0.70–2.52)	0.387
Fourth quartile	1.81 (1.02–3.22)	0.044*	1.62 (0.83–3.17)	0.160
Calcaneus				
Second quartile	1.67 (0.92–3.02)	0.092	1.71 (0.91–3.20)	0.094
Third quartile	1.18 (0.63–2.22)	0.604	1.12 (0.57–2.22)	0.737
Fourth quartile	1.62 (0.89–2.94)	0.113	1.50 (0.75–3.00)	0.253

a Adjusted for age, sex, race, smoking, and coffee and alcohol consumption.

* Statistically significant.
